# Carbonate Apatite Nanoparticles-Facilitated Intracellular Delivery of siRNA(s) Targeting Calcium Ion Channels Efficiently Kills Breast Cancer Cells

**DOI:** 10.3390/toxics6030034

**Published:** 2018-06-26

**Authors:** Mohammad Borhan Uddin, Balakavitha Balaravi Pillai, Kyi Kyi Tha, Maeirah Ashaie, Md. Emranul Karim, Ezharul Hoque Chowdhury

**Affiliations:** 1 Faculty of Medicine, Nursing and Health Sciences, Monash University, Wellington Rd & Blackburn Rd, Clayton, VIC 3800, Australia; bnn.nsu@gmail.com (M.B.U.); brav2@student.monash.edu (B.B.P.); tha.kyi.kyi@monash.edu (K.K.T.); maira.ashaie@gmail.com (M.A.); karim604306@gmail.com (M.E.K.); 2 Department of Pharmaceutical Sciences, School of Health and Life Sciences, North South University, Dhaka 1229, Bangladesh

**Keywords:** carbonate apatite, siRNA, MCF-7 breast cancer cell, cytotoxicity, ion channels and transporter genes

## Abstract

Specific gene knockdown facilitated by short interfering RNA (siRNA) is a potential approach for suppressing the expression of ion channels and transporter proteins to kill breast cancer cells. The overexpression of calcium ion channels and transporter genes is seen in the MCF-7 breast cancer cell line. Since naked siRNA is anionic and prone to nuclease-mediated degradation, it has limited permeability across the cationic cell membrane and short systemic half-life, respectively. Carbonate apatite (CA) nanoparticles were formulated, characterized, loaded with a series of siRNAs, and delivered into MCF-7 and 4T1 breast cancer cells to selectively knockdown the respective calcium and magnesium ion channels and transporters. Individual knockdown of *TRPC6*, *TRPM7*, *TRPM8*, *SLC41A1*, *SLC41A2*, *ORAI1*, *ORAI3*, and *ATP2C1* genes showed significant reduction (*p* < 0.001) in cell viability depending on the cancer cell type. From a variety of combinations of siRNAs, the combination of TRPC6, TRPM8, SLC41A2, and MAGT1 siRNAs delivered via CA produced the greatest cell viability reduction, resulting in a cytotoxicity effect of 57.06 ± 3.72% (*p* < 0.05) and 59.83 ± 2.309% (*p* = 0.09) in 4T1 and MCF-7 cell lines, respectively. Some of the combinations were shown to suppress the Akt pathway in Western Blot analysis when compared to the controls. Therefore, CA-siRNA-facilitated gene knockdown in vitro holds a high prospect for deregulating cell proliferation and survival pathways through the modulation of Ca^2+^ signaling in breast cancer cells.

## 1. Introduction

The cell membrane is a lipid bilayer containing thousands of membrane proteins that control numerous cell signal-transduction pathways. Some membrane proteins also control a huge range of gradients to achieve chemical, electrical, and mechanical homeostasis of the cell. The steady-state maintenance of highly asymmetric concentrations of the major inorganic cations (Na^+^, K^+^, Ca^2+^, H^+^, Mg^2+^) and anions (Cl^−^, phosphate, bicarbonate) is a critical function carried out by the plasma membrane as well as the membranes of intracellular organelles [1]. Ion channels have been shown to be dysregulated (under-expressed or overexpressed) in various cancers, namely prostate, hepatocellular, and non-estrogen-sensitive breast cancers, leading to enhanced drug efflux, reduced drug influx, and failure of apoptosis, thus strengthening tumorigenesis [2,3].

Despite rapid development in the past 25 years in exploring the ion channel functions in relation to cancer, most of the mechanisms accounting for the impact of ion channel modulators on cancer growth have yet to be fully clarified. However, numerous in vivo experiments targeting ion channels in various cancer models demonstrated the great potential of this approach, highlighting ion channels as viable oncological targets [4,5,6].

Calcium ions (Ca^2+^) play a vital role as second messengers in gene expression, cell cycle control, migration, survival, and apoptosis [7]. They play numerous roles in cell signaling pathways, most abundantly in the protein kinase pathways. The correlation of calcium influx leading to reduced cell proliferation and increased cell death has been gaining momentum to be an established point of control in cell pathways, among few, the MAPK (mitogen-activated protein kinases) and AKT (protein kinase B) pathways. In breast cancer, aberrant Ca^2+^ signaling and intracellular Ca^2+^ homeostasis have been proposed as crucial events in tumorigenesis. Dysregulated calcium influx and efflux has been shown to enhance survival, malignant angiogenesis, excessive proliferation, cell migration, and metastasis in cancer cells [2]. Indeed, the Ca^2+^ signaling “toolkit” consisting of a number of Ca^2+^ transport molecules and ancillary proteins has been suggested as a therapeutic target in cancer [5,8,9,10,11,12]. Other than a dysregulated fashion of calcium signaling, calcium flux promotes the cell proliferation, migration, metastasis, and survival of breast cancer [8,9,10].

Ca^2+^ channels in both intracellular stores (endoplasmic reticulum (ER) and mitochondria) and extracellular space [7] are categorized into (i) voltage-gated/voltage-dependent Ca^2+^ channels (CaV) and (ii) non-voltage-gated/voltage-independent Ca^2+^ channels, which are again sub-divided into the TRP, ORAI, and STIM subfamilies [7,13]. TRP channels (especially TRPM7, a calcium and magnesium ion permeable channel) have been heavily researched on their upstream properties in pathology, especially malignancy [2,14]. *ORAI* genes encode the calcium release-activated calcium channel proteins and the knockdown of *ORAI1* genes directly reduces proliferation in MCF-7 cells [14]. Upon the depletion of intercellular calcium ions, a synergistic effect between the endoplasmic reticulum calcium channels and the ORAI proteins induces calcium influx [13,15] and plays a pivotal upstream role in cancer proliferation [15,16]. In breast cancer, *ORAI3* knockdown might halt cancer mitosis, eventually inhibiting cancer proliferation as well. Thus, the silencing of *ORAI1* and *ORAI3* expression seems important in arresting all tumorigenesis processes [15,17,18]. In the MCF-7 cell lines, *TRPC6* is highly expressed compared to the adjacent non-tumor cells. TRPV6 is expressed at elevated messenger levels in breast cancer patient samples. *TRPV6* might not only maintain an increased proliferation rate, but also increases cellular survival and provides resistance to apoptosis [15].

The development of efficient siRNA nanocarriers for systemic delivery represents an important goal in cancer therapy. The properties of an ideal nanoparticle (NP) for this purpose would include the capacity to remain in the circulation, deliver the siRNA payload to the target organ, interact with the cell surface, enter the cell, and, finally, efficiently escape the endosome–lysosome system to unload the siRNAs within the cytoplasm [19,20]. Carbonate apatite (CA) nanocrystals are basically made of an inorganic cation (calcium) and inorganic anions, namely, carbonate and phosphate possess both anion- and cation-binding domains, enabling them to bind to negatively charged siRNA molecules [21].

Very few studies have been conducted so far on the roles of calcium ion channels and transporters in the survival and proliferation of breast cancer cells. Here we aimed to develop smart NP-siRNA complexes as a therapeutic tool for breast cancer by efficiently delivering the specific siRNAs against calcium ion channels and transporter genes using CA nanoparticles (NPs) in order to suppress the expression of each of the transporter proteins and evaluate their potential as therapeutic targets in the MCF-7 breast cancer cell line.

## 2. Materials and Methods

### 2.1. Reagents

Dulbecco’s modified Eagle medium (DMEM) was purchased from BioWhittaker (Walkersville, MD, USA). DMEM powder, fetal bovine serum (FBS), and trypsin-ethylenediamine tetraacetate (trypsin-EDTA) were obtained from Gibco BRL (San Francisco, CA, USA). Calcium chloride dehydrate (CaCl_2_·2H_2_O), sodium bicarbonate, dimethyl sulphoxide (DMSO), and thiazolyl blue tetrazolium bromide (MTT) were bought from Sigma-Aldrich (St. Louis, MO, USA). Human breast cancer cell line MCF-7 (ATCC^®^ HTTB-22 TM Homo sapiens mammary gland) was used. The AF 488 negative siRNA and all functionally validated siRNAs (Table 1) used in this study against calcium ion channels and transporter genes were from QIAGEN (Valencia, CA, USA). Polyclonal antibodies for human p42/44 Erk, pan-Akt, phospho-Akt, and phospho-p42/44 Erk were purchased from Cell Signaling Technology (CST), Massachusetts. Proteins raised in rabbit were procured from Thermo Scientific.

### 2.2. Design and Reconstitution of siRNAs

The functionally validated siRNA(s) with the target sequences (Table 2) were supplied in lyophilized form and upon delivery each was reconstituted with RNase-free water to obtain a stock solution of 20 μM. The siRNA solution was then allocated into multiple reaction tubes for storage as repeated thawing might affect the silencing efficiency of siRNA. The siRNAs were stored at −20 °C as recommended by Qiagen.

### 2.3. Preparation of CA NPs

NPs were formed in freshly prepared bicarbonated (44 mM) DMEM media (pH 7.4) by mixing exogenous CaCl_2_ (1–6 µL from 1 M stock), followed by incubation of the mixture for 30 min at 37 °C. Finally, media was added to top up the volume of the particle suspension to 1 mL, and FBS was added to all samples to prevent aggregation. To ensure the appropriate size of the NPs, the absorbance of the particle suspension was measured immediately after fabrication at 320 nM using a spectrophotometer (UV 1800 Spectrophotometer, Shimadz, Kyoto, Japan), as an indicator of turbidity which increases with an increase in particle number and size [21].

### 2.4. Preparation of siRNA-Bound CA NPs

NPs were prepared as described in Section 2.3 in freshly prepared bicarbonated (44 mM) DMEM media (pH 7.4) by mixing 3 to 4 µL of 1 M CaCl_2_, followed by incubation of the mixture for 30 min at 37 °C. For making complexes with siRNA(s) (NPs-siRNA(s)), 1 nM of allstars negative control siRNA or 1–2 nM of a functionally validated single siRNA was added to 200 µL DMEM media along with the appropriate amount of CaCl_2_ prior to 30 min of incubation at 37 °C. To fabricate NP complexes of fluorescent negative siRNA, 10 nM of allstars negative control siRNA AF488 was used along with 3 µL of 1 M CaCl_2_, for the studies of siRNA binding to NPs and cellular uptake of the siRNA. After 30 min of incubation at 37 °C, media was added to top up the volume to 1 mL, with 10% FBS added subsequently.

### 2.5. Measurement of Particle Size of NPs

Size of the NPs formed in different concentrations of CaCl_2_ exogenously added for induction of solution supersaturation was measured using Zetasizer (Nano ZS, Malvern, UK) after adding 10% FBS and temporarily keeping on ice to prevent particle aggregation. A refractive index ratio of 1.325 was set for the estimation of the particle diameter. Data were analyzed using Zetasizer software 6.20 and all samples were measured in duplicate.

### 2.6. Microscopic Observation of Aggregated and Nano-Sized Particles

Using an optical microscope and subsequently a field emission scanning electron microscope (FE-SEM), aggregated and nano-sized crystals were measured, respectively, for the subsequent studies. The aggregation of particles was visualized as large-sized crystals under an optical microscope. Particles were prepared in 200 µL of freshly prepared bicarbonated (44 mM) DMEM media (pH 7.4) by mixing 3–6 µL from 1 M CaCl_2_, followed by incubation of the mixture for 30 min at 37 °C, as described in detail in Section 2.3.

NPs prepared in 200 µL DMEM media with 15 mM of CaCl_2_ was visualized through a field emission scanning electron microscope (HitachiS-4700 FE-SEM, Tokyo, Japan). NPs were centrifuged at 15,000 rpm for 10 min, followed by the removal of the supernatant and resuspension with milli-Q water. Prepared NP suspensions were maintained under ice before microscopic observation. One microliter of each sample was placed onto a carbon tape-coated sample holder and dried at room temperature, followed by platinum sputtering of the dried samples for 30 s. Microscopic observation of the sputtered nanoparticles was conducted using FE-SEM at 5–10 kV.

### 2.7. Cell Culture and Seeding

MCF-7 cells were grown in a 25-cm^2^ culture flask in DMEM supplemented with 10% heat-inactivated FBS in a humidified atmosphere containing 5% CO_2_ at 37 °C. Exponentially growing MCF-7 cells were trypsinized and, following the addition of fresh medium, the cell suspension was centrifuged at 10,000 rpm for 5 min and the supernatant was discarded. Fresh medium was added to resuspend the pellet and the cells were counted using a hemocytometer. Appropriate dilutions were made using culture medium to produce a cell suspension with a concentration of 5.0 × 10^4^ cells/mL. One milliliter of the prepared cell suspension was subsequently added to each of the wells in a 24-well plate and allowed to grow overnight at 37 °C and 5% CO_2_ before siRNA transfection.

### 2.8. Binding Affinity of siRNA towards CA NPs

Particles with nano-size dimensions were investigated on the basis of their abilities to bind fluorescence-labeled siRNA, using both a fluorescence microscope and a fluorescence plate reader. Fluorescence-labeled siRNA/nanoparticles were incubated with the MCF 7 cells for 1–4 h prior to the qualitative and quantitative studies. Particles with high affinity to siRNA molecules and potential capacity for cellular endocytosis (as predicted from the estimation of their sizes) were selected for the intracellular delivery of siRNA and subsequent cytotoxicity study. NPs were prepared with different concentrations of Ca^2+^ (as previously mentioned in Section 2.3). The formed CA-siRNA complexes were centrifuged at 13,000 rpm for 15 min. The resulting pellets were dissolved with the addition of 20 mM EDTA in PBS. One hundred microliters of each sample was placed into the 96-wells plate by Opti-plate (Nunc) and the fluorescence intensity was measured by using the 2030 multilabel reader vitor™ X5 (Perkin Elmer, Waltham, MA, USA) and analyzed via Perkin Elmer 2030 manager software with λex = 490 nM and λem = 535 nM. The calibration was made using different concentrations of free fluorescence-labeled siRNA (Figure S1).

### 2.9. Cellular Uptake of Fluorescence-Labeled siRNA Carried by CAs

To observe the cellular uptake, 10 nM of AF 488 neg. siRNA was complexed with NPs formed with a fixed concentration of Ca salt, as described in Section 2.3. After 30 min of incubation at 37 °C, 10% FBS-containing DMEM was added to the nanoparticles formulation to stop particle aggregation. MCF-7 cells were treated with these NP-bound siRNA. After 2 h, cells were washed with 5 mM EDTA in PBS to remove extracellular NPs and siRNA-bound NPs and observed under a fluorescence microscope. The fluorescence intensity of the treated cells was measured with an excitation at 485 nM and an emission at 535 nM using PerkinElmer 2030 manager software attached to a 2030 multilabel reader victor X5 (PerkinElmer, Waltham, MA, USA).

### 2.10. Cell Viability Assessment with 3-(4,5-Dimethylthiazol-2-yl)-2,5-diphenyltetrazolium Bromide (MTT) Assay

MCF-7 cells were seeded on a 24-well plate with a density of 5 × 10^4^ cells/well in 1 mL DMEM (high glucose) supplemented with 10% FBS and a 1% penicillin-streptomycin solution. After incubation at 37 °C in a humid environment of 5% CO_2_ for 24 h, cells were treated with CA NPs prepared with different concentrations of Ca^2+^ and 1 nM of siRNA (either allstars negative control siRNA or a functionally validated siRNA) in 1 ml of DMEM and incubated for 48 h. The untreated cells served as a blank control. The fraction of viable MCF-7 cells was determined using MTT assay. Briefly, 50 μL of MTT (5 mg/mL in PBS) was added aseptically into each of the treated wells, followed by incubation at 37 °C and 5% CO_2_ for 4 h. After incubation, the medium containing MTT was aspirated and the purple formazan crystals at the bottom of each well were dissolved by mixing with 300 μL of DMSO solution. The absorbance of the resulting formazan solution was then determined spectrophotometrically at wavelength 595 nM using a microplate reader (Dynex Opsys MR™, Tarporley, UK) with reference to 630 nM. Each experiment was performed in triplicate and the data were plotted as the mean ± standard deviation (SD) of three independent experiments.

### 2.11. Western Blot Analysis

Cells treated with CA NPs carrying one or more functionally validated siRNAs and incubated for 48 h were lysed with IP lysis buffer and subjected to centrifugation at 13,000 rpm for 20 min at 4 °C. The supernatant, comprising a protein sample, was collected and 5 µL was aspirated to estimate the total amount of proteins through a bovine serum albumin (BSA) assay kit based on the manual. In the initial step, BSA protein was used to create the standard curve, which was used to calculate the total protein concentration of cellular lysates based on their absorbance intensity. The remaining samples were aliquoted and stored at −80 °C for subsequent SDS-PAGE and Western blot analyses. The cellular lysates containing 30 µg of total protein were mixed with 10 µL of 10× loading dye and subjected to SDSPAGE using stain-free mini protein SFX gels (15 wells) in 1× running buffer at 0.01 amp/gel. Seven microliters of precision plus protein standards-dual color was used as a molecular weight marker to establish the molecular weight of the sampled proteins. The transfer of protein samples from the gel to the 0.2 µm PVDF membranes attached to a trans-blot turbo transfer pack through a trans-blot turbo transfer system was performed for 7 min at 1.3 amp, followed by blocking in 5% skimmed milk in 1× TBST for 1 h at room temperature. The membrane was next incubated with primary antibodies (pMAPK, TMAPK, pAKT, TAKT, and GAPDH as a loading reference) at 4 °C overnight with gentle shaking, followed by washing in 1× TBST five times to remove unbound primary antibodies. HRP-conjugated goat anti-rabbit secondary antibody Ig G (1:3000) was introduced into the membrane for 1 h with mild agitation, before washing five times in 1× TBST to again eliminate the unbound antibodies. The membrane was exposed to a mixture of ECL for 5 min before the observation of bands through chemiluminescence signals using an XRS Chemidoc system (BioRad, Hercules, CA, USA).

### 2.12. Data Analysis

The cell viability in the treated wells was expressed as a percentage and was calculated using the absorbance values obtained from the MTT assay by using the following formula:

$$\%\;\mathrm{cell}\;\mathrm{viability}\;=\frac{\mathrm{Absorbance}\;\mathrm{of}\;\mathrm{treated}\;\mathrm{sample}}{\mathrm{Absorbance}\;\mathrm{of}\;\mathrm{control}}\times 100\%$$

Subsequently, the cytotoxicity percentage was calculated by subtracting the percent cell viability of siRNA-loaded NPs from the percent cell viability of free CA nanoparticles

### 2.13. Statistical Analysis

Statistical analysis was conducted using the SPSS statistical package (version 17.0 for Windows). LSD post-hoc test for one-way ANOVA was used to analyze and compare the significant difference between treated and non-treated samples. Data are presented as the mean ± SD with *p* < 0.05 being considered as statistically significant.

## 3. Results and Discussion

### 3.1. Optimization of CA NPs Based on Turbidity, Particle Size, and Cytotoxicity Profiling

**Figure 1:**
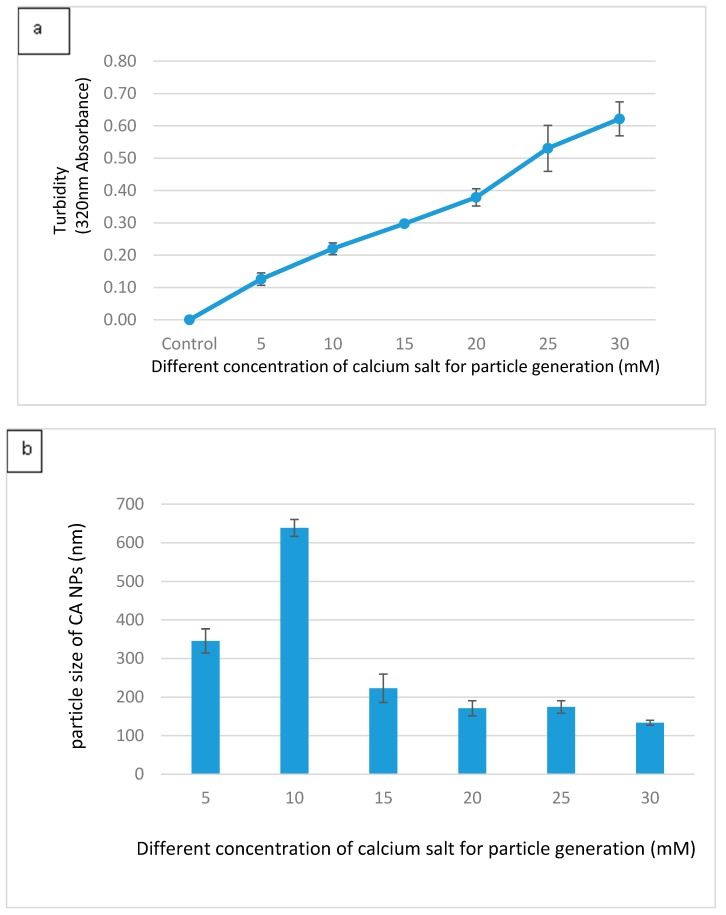
(**a**) Measurement of turbidity at 320 nM as an indicator of particle growth, which was induced by the addition of different concentrations of Ca^2+^ to 200 µL bicarbonate-buffered solution (pH 7.4), followed by incubation at 37 °C for 30 min. The data are presented as the mean ± SD of triplicates. (**b**) Particle sizes of CA NPs formed in the presence of varying concentrations of Ca ions. Fabricated particles were analyzed using Zetasizer to obtain the average size. The data are presented as the mean ± SD of triplicates.

**Figure 2:**
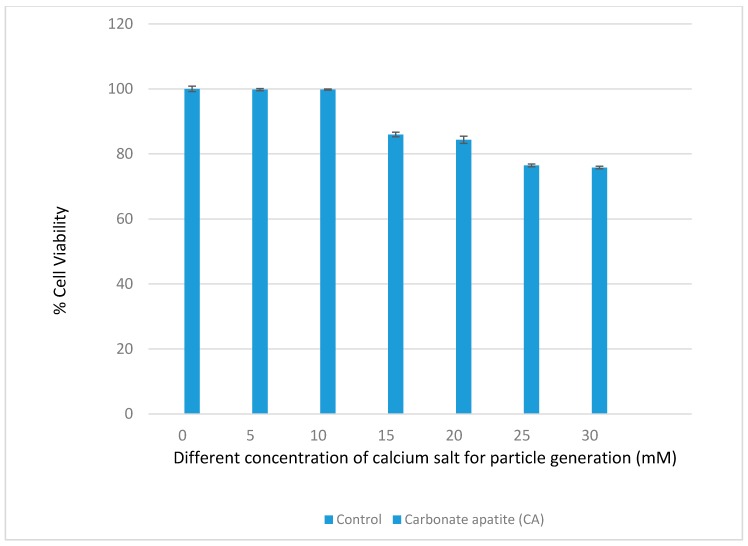
Assessment of the cytotoxicity of CA nanoparticles (NPs) in MCF-7 cells. MCF-7 cells were incubated for a consecutive period of 48 h with the particles formulated with different concentrations of calcium chloride and subjected to assessment for cell viability by MTT assay. The data are presented as the mean ± SD of triplicates.

Different formulations of CA were made with different concentrations of Ca^2+^ in 200 µL DMEM containing fixed amounts of inorganic phosphate and bicarbonate in order to find out the suitable formulation for intracellular delivery of the target siRNAs. As shown in Figure 1a, an increasing trend in absorbance was observed with increasing concentrations of Ca^2+^, indicating that the turbidity, i.e., the growth of the formulated CA particles, was enhanced when the concentration of Ca^2+^ increased. Figure 1b shows that the average size of the particles developed in 200 µL DMEM gradually decreased with increasing concentrations of Ca^2+^. When the concentration of Ca^2+^ reached 30 mM, the particles were in the smallest range of diameter. The depletion of inorganic phosphate which was present at 0.9 mM in the medium probably resulted in the slow growth of the particles without a further increase in the average particle diameter. Thus, 3–6 µL of 1 M exogenous calcium chloride (i.e., 15–30 mM) is necessary to induce particles with an average size range of 125–225 nM in 200 µL DMEM. Interestingly, in our earlier studies particle fabrication in 1 mL DMEM led to an increase in both turbidity and particle diameter upon an increase in the concentration of exogenous Ca^2+^ (1–6 µL of 1 M Ca^2+^), with the highest particle size observed at 6 mM of Ca^2+^ [22,23,24]. This could be explained by the notion that a relatively lower concentration of exogenous Ca^2+^ (1–6 mM) in 1 mL DMEM, unlike 200 µL DMEM with 5–30 mM of exogenous Ca^2+^, is unable to react all inorganic phosphate (Pi) in the solution, with the remaining Pi stimulating further growth and consequently the aggregation of particles. This particularly occurred with the concentration 6 mM of Ca^2+^, which induced the generation of more particles. As a matter of fact, when the cells were treated with the particles formed with 6 mM of exogenous Ca^2+^ in 1 mL DMEM, the cells appeared to be surrounded by and overloaded with the aggregated particles under an optical microscope, leading to potential cytotoxicity with cell viability observed at less than 50%. However, as shown in Figure 2 of our current study, nanoparticles formulated with 6 µL of 1 M Ca^2+^ in 200 µL of DMEM exhibited a much lower cytotoxicity to MCF-7 cells compared to our earlier studies. Smaller sized CA NPs are more efficiently taken up by the treated cells through endocytosis than the larger ones, exerting less toxicity to the cells [25].

**Figure 3:**
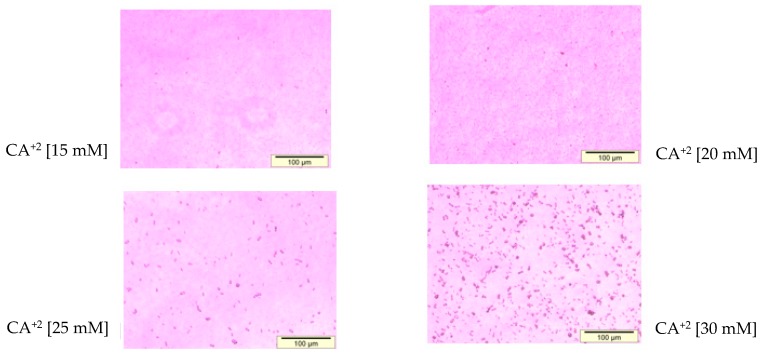
Optical microscopic size estimation of NPs developed by mixing 15–30 mM of exogenous CaCl_2_ (3–6 µL from 1 M stock), followed by incubation of the mixture for 30 min at 37 °C, as described in detail in Section 2.3. The aggregation of the particles was visualized by the observation of large-sized crystals under an optical microscope. All images were captured at 10× resolution.

**Figure 4:**
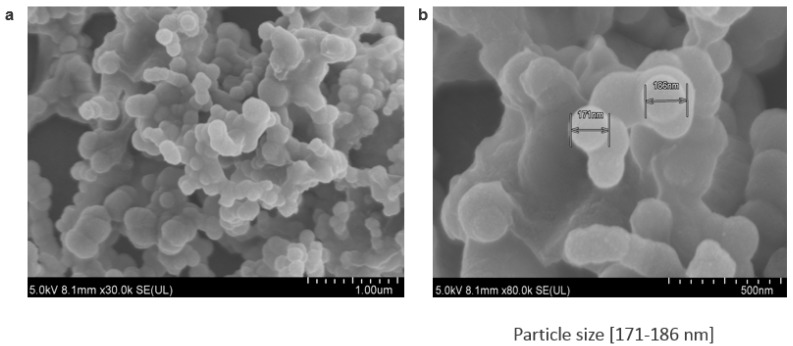
The size and morphology of various nanocrystal samples visualized through FE-SEM (field emission scanning electron microscope; HitachiS-4700 FE-SEM, Japan). Particle aggregations (**a**) and individual particles size (**b**) were observed.

Through observation under an optical microscope (Figure 3), the aggregated particles prepared with 1–6 µL of 1 M Ca^2+^ as well as the particles formed with 3–4 µL of 1 M exogenous calcium chloride were found to demonstrate mild to moderate aggregation. FE-SEM images of the particles fabricated with 3 µL of 1 M Ca^2+^ (Figure 4) had a particle size in the range of 171–186 nM, which is slightly smaller than that of the same particles (191–284 nM) measured with Zetasizer (Table 3).

### 3.2. Binding Efficiency of Fluorescence-Labeled siRNA-CAs Complex

**Figure 5:**
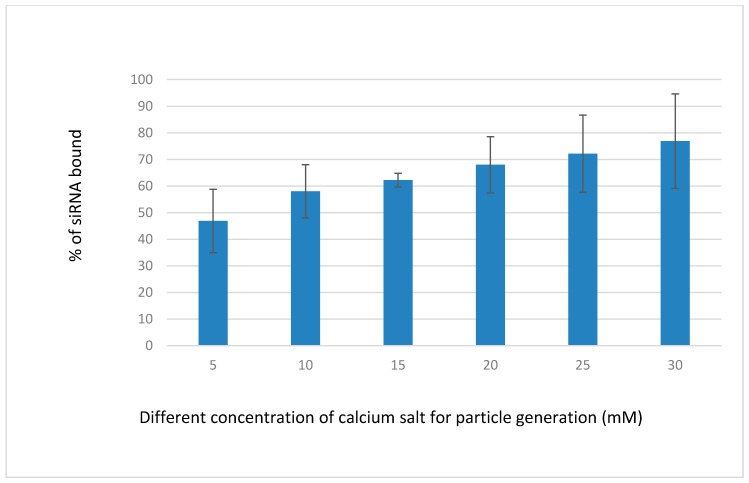
Binding efficiency of fluorescence-labeled siRNA-CAs complex developed by mixing 5–30 mM of exogenous CaCl_2_ (1–6 µL from 1 M stock), followed by incubation of the mixture for 30 min at 37 °C, as described in detail in Section 2.3. The concentration of the allstars negative siRNA (AF 488) (QIAGEN) (fluorescence siRNA) was 10 nM.

Binding affinity of NPs towards siRNA was performed to confirm the experimental design with sufficient loading capacity. Fluorescent negative sRNA (10 nM) was used to check the difference of interactions between the siRNA and the NPs formed with increasing concentrations of CaCl_2_ (Figure 5), while keeping other reactants, inorganic phosphate, and bicarbonate constant for particle formation. The interaction efficiency of siRNA with NPs was steady with 15 mM of Ca^2+^, which might be due to the uniform size distribution and moderate aggregation of the particles. Negative charges on the phosphate backbone of siRNA could bind very efficiently to the Ca^2+^-rich domains of the carbonate apatite NPs through ionic interactions [22].

### 3.3. Cellular Uptake Study of CA NPs by Fluorescence Microscopy

**Figure 6:**
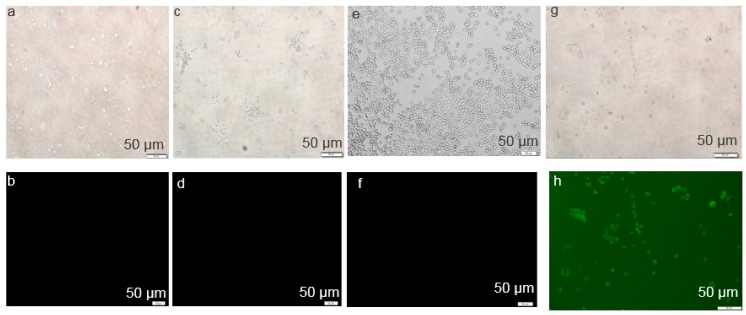
Cellular uptake of NP-bound fluorescent negative siRNA. MCF-7 cells were treated with (**a**,**b**) media (untreated), (**c**,**d**) NPs, (**e**,**f**) free siRNA, and (**g**,**h**) NPs-siRNA formed with 15 mM of CaCl_2_ and 10 nM AF488 negative control siRNA. All images were captured at 10× resolution.

The successful internalization of NPs-siRNA by cells with the subsequent release of free siRNA in cytosol is the most crucial step for the effective silencing of target mRNA. As shown in Figure 6, the cellular uptake of NPs-siRNA formed with 3 mM of exogenous CaCl_2_ was compared with untreated/NP-treated and free siRNA-treated cells. The corresponding light photographs (top panel) showed the density of cells in a particular area used to capture fluorescence images (bottom panel). Cells that were kept untreated or treated with NPs or free siRNA did not show any fluorescence (Figure 6b,d,f) under the microscope after washing out extracellular particles with EDTA. On the other hand, cells treated with NPs-siRNA showed a diffusive pattern of fluorescence (Figure 6h) after the washing, thus confirming the internalization of NPs-siRNA in breast cancer cells, as shown previously in breast tumor studies [22,26]. In this study, CA NPs with smaller particle sizes seemed to be capable of carrying electrostatically associated siRNA inside cancer cells through endocytosis, eventually releasing the bound siRNA from the endosome to the cytosol through pH-responsive self-dissolution to induce death in breast cancer cells [22,23].

### 3.4. Cell Viability Assessment Following Intracellular Delivery of Individual siRNAs with CA NPs

**Figure 7:**
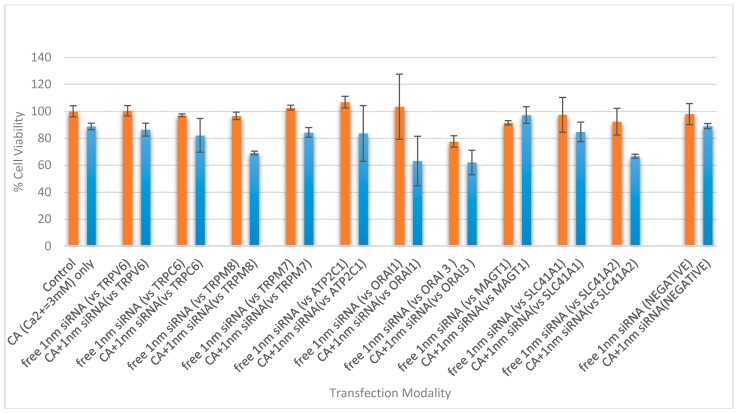
Cell viability assessment of MCF-7 cells treated with NP-siRNA complex and unbound siRNA, where the NP is CA formed with 4.0 mM exogenous CaCl_2_. The cells were treated with media (negative control) or NPs formed with 4.0 mM of exogenous CaCl_2_ (CA only), and were compared with different siRNA against cation channels and transporter proteins, of 1 nM concentration, both free and bound to the inorganic NP. After 44 h of treatment, cell viability was measured by MTT assay. Values are represented as the mean % of cell viability ± SEM, when compared to untreated cells, where N = 3. Values are presented as the mean ± SD of % of cell viability compared to untreated cells for triplicate samples.

There was an obvious decrease in cell viability when functionally validated siRNAs (1 nM) targeting TRPM8 and SLC41A2 were individually delivered into MCF-7 cells using NPs in comparison to the respective naked siRNA, whereas no statistically significant variation in cytotoxicity was observed between negative siRNA-loaded NPs and NPs alone (Figure 7). TRPM8, a cation-permeable ion channel, was found to be highly expressed in various breast cancer sub-types [27,28], promoting their metastasis by regulating EMT via activating the AKT/GSK-3β pathway [29]. On the other hand, SLC41A2 is a plasma-membrane Mg^2+^ transporter expressed in vertebrate cells [30]. The decrease in cell viability as a result of silencing the *SLC41A2* gene sheds light on its potential roles in the survival or proliferation of MCF-7 breast cancer cells. Despite the fact that other cation transporters are also expressed in MCF-7 cells, the inconsistent results obtained with their respective siRNAs in inducing cell death have yet to be fully understood. However, in an independent experiment, intracellular delivery of siRNA (1 nM) targeting *ATP2C1*, *TRPM7* or *ORAI3* using NPs (formulated with 3 µL of 1 M CaCl_2_ in 200 µL of DMEM) led to a statistically significant reduction in cell viability in MCF-7 cells (Figure S2).

**Figure 8:**
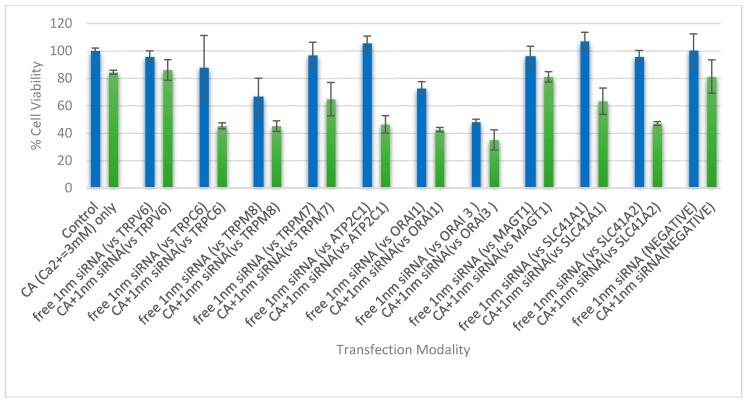
Cell viability assessment of 4T1 cells treated with NP-siRNA complex and unbound siRNA, where the NP is CA formed with 4.0 mM exogenous CaCl_2_. The cells were treated with media (negative control) or NPs formed with 4.0 mM of exogenous CaCl_2_ (CA only), and were compared with different siRNA against cation channels and transporter proteins, of 1 nM concentration, both free and bound to the inorganic NP. After 44 h of treatment, cell viability was measured by MTT assay. Values are represented as the mean % of cell viability ± SEM, when compared to untreated cells, where N = 3.

In 4T1 cells, however, except TRPV6 and MAGT1 siRNAs, all siRNAs targeting TRPC6, TRPM7, TRPM8, SLC41A1, SLC41A2, ORAI1, ORAI3, and ATP2C1 showed a decline in cell viability when complexed with CA NPs, compared to naked siRNA and NPs alone (Figure 8). TRPC6 was found to be overexpressed in mRNA and protein levels in breast carcinoma specimens in comparison to normal breast tissue, indicating its potential role in breast carcinogenesis [31], whereas TRPM7 regulates proliferation, migration, invasion, and metastasis of breast cancer cells depending on the stimulation of their ion channels and kinase domains [32,33] and TRPM8 is involved in the initiation and progression of tumors with its aberrant expression found in varieties of tumors including breast adenocarcinoma [34]. The Na^+^/Mg^2+^ exchanger SLC41A1 (A1) is the major cellular Mg^2+^ efflux system, which, when overexpressed, decreases intracellular Mg^2+^ and influences the activity of kinases of anti-apoptotic and pro-survival pathways [35]. ORAI1 and ORAI3 are plasma membrane-embedded calcium channels having roles in cellular calcium homeostasis by allowing Ca^2+^ to enter and refill the sarcoplasmic/endoplasmic reticulum Ca^2+^ stores [36]. Alteration in the expression of ORAI proteins was shown to be correlated to the onset and maintenance of tumor phenotype in many solid malignancies including breast cancer [37]. ATP2C1 plays an important role in maintaining low cytoplasmic Ca^2+^ levels at rest and priming organellar stores and high levels of Ca^2+^ for a wide range of signaling functions [38]. Dysregulation in Ca(^2+^)-ATPase expression was reported in cancers of the colon, lung, and breast [38].

In our previous studies, we showed that pH-sensitive CA efficiently delivered nucleic acids, such as plasmid DNA and siRNA, into the mammalian cells by virtue of its high affinity interactions with DNA or siRNA and the desirable size of the resulting complexes for the effective cellular endocytosis. Moreover, following internalization by cells, the nucleic acid molecules were found to be released from both the particles and the endosomes, as a result of particle dissolution during endosomal acidification. [21,22,39]. The suppression of the cation channels and transporters with their respective siRNAs might result in the breakdown in Ca^2+^ and Mg^2+^ homeostasis, leading to dysregulated calcium and magnesium signaling [40,41]. The dysregulation of Ca^2+^ homeostasis was probably further aggravated by the intracellular, pH-dependent dissolution of CA NPs, leading to a calcium overload that could trigger cell death [42,43].

### 3.5. Cell Viability Study with CA-Multiple siRNAs

**Figure 9:**
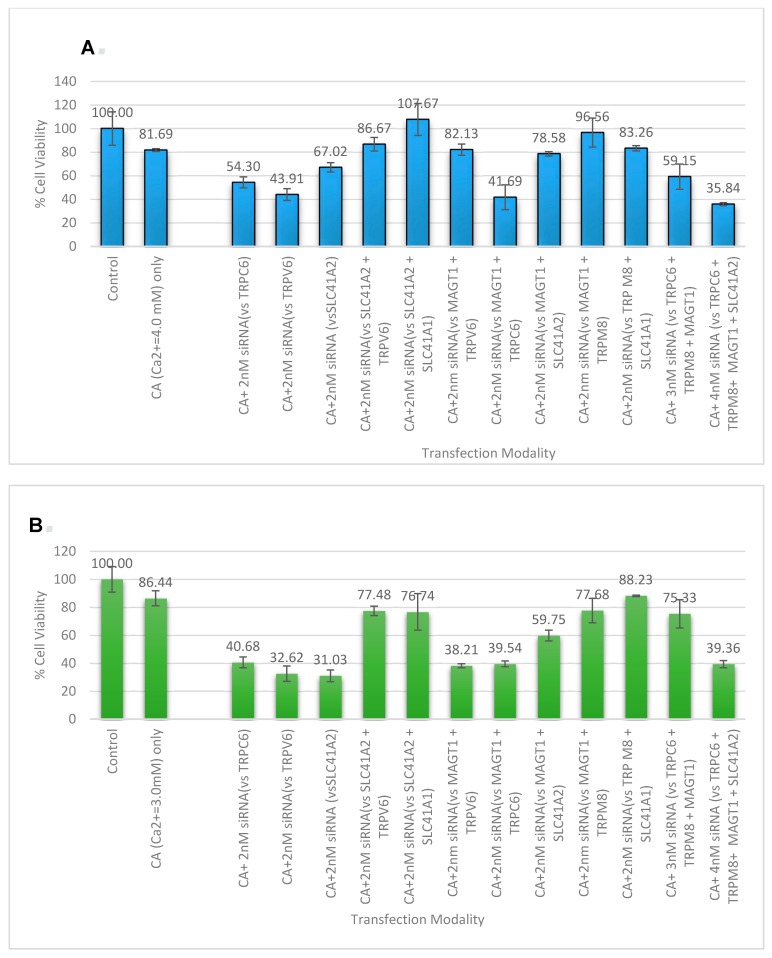
CA-multiple siRNAs to determine synergistic cytotoxicity: (**A**) MCF-7 cells; (**B**) 4T1 cells. Cell viability assessment in breast cancer cells for the NP-siRNA formed with a fixed concentration of exogenous CaCl_2_. Cells were treated with media (untreated), NPs formed with 3 mM (for 4T1) and 4 mM (for MCF-7) of exogenous CaCl_2_, and different combinations of NP-siRNA formed with 1 nM of individual siRNA. After 44 h of treatment, cell viability was measured by MTT assay. Values are represented as the mean % of cell viability ± SEM, when compared to untreated cells, where N = 3.

CA-bound siRNAs of different combinations of the three magnesium transporter proteins (MAGT1, SLC41A1, and SLC41A2) along with other calcium receptor channels and transporters (TRPV6, TRPC6, and TRPM8) were fabricated, where individual siRNAs targeting six single genes were used at a concentration of 2 nM, and siRNAs for every couplet combination (30), trio combination (13), quad combination (30), five siRNA combination (2), and a mix of all six siRNAs (a total 49 combinations) were added at 1 nM (Figure 9). Transfection was carried out on MCF-7 and 4T1 and MTT assay was repeated on all of the combinations of siRNAs that were shown to significantly lower cell viability compared to untreated and NP-Ca treated cells, as is represented in Figure 9A,B. The cell viability reduction across the cell line is highly consistent when the single genes *TRPV6*, *TRPC6*, and *SLC41A2* were subjected to knockdown. Genes in combination with the magnesium transporter, MAGT1, did not show much reduction in cell viability until synergistically silenced along with TRPC6. The greatest reduction in cell viability was observed when the knockdown of four genes, *TRPC6*, *TRPV6*, *MAGT1*, and *SLC41A2*, was carried out via CA-mediated siRNA delivery, resulting in the most prominent effect on MCF-7 with only 35.84 ± 1.265% cell viability, and 39.36 ± 2.583% cell viability of 4T1.

We infer that this is probably because there are more than one type of calcium or magnesium channels for the cells, thus the knockdown of a group of these proteins is necessary to produce a robust effect. As the greatest synergistic effect was seen when a magnesium transporter (MAGT1) was suppressed along with the other three main proteins, it is highly likely that this divalent cation transporter is not solely responsible for magnesium influx [3,44]. Interestingly, increasing the siRNA concentration to 2 nM promoted significant cytotoxic effects in MCF-7 with the siRNAs (Figure 9) which did not show cytotoxicity at 1 nM concentration (Figure 7), indicating that siRNA concentration is one of the parameters influencing the therapeutic outcome in vitro.

### 3.6. Effects of NP-Facilitated Delivery of siRNAs against Cation Channels and Transporters on MAPK and AKT Activation in MCF-7 Cells

The activation levels of mitogen-activated protein kinase (MAPK) and protein kinase B (PKB), better known as AKT, signal transduction pathways were determined using protein analysis via Western blot. Cellular lysates of MCF-7 cells that were treated with six CA-siRNA complexes—which exhibited the greatest cytotoxicity effects, as shown in Figure 9—were used. The proteins analyzed were both phosphorylated and unphosphorylated (total) forms of MAPK and AKT. The housekeeping protein used for qualitative normalization was GAPDH.

Since the knockdown of TRPC6 resulted in a very high cytotoxic effect (Figure 9), TRPC6 siRNA was combined with other siRNAs that also demonstrated effective reduction in cell viability to see if there was a synergistic effect on the MAPK and AKT pathways, both of which involve calcium signaling eventually affecting cell proliferation and apoptosis.

**Figure 10:**
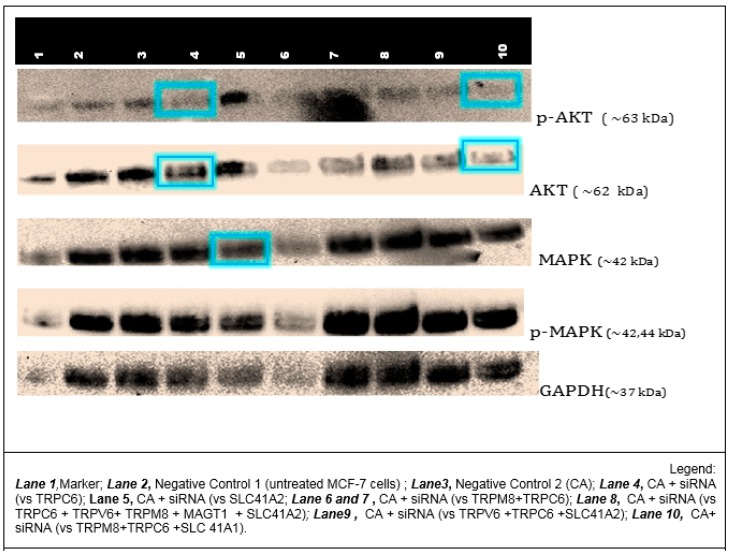
Western blot analysis for 42/44-ERK (total MAPK) and total AKT and their phosphorylated forms following treatments with NP-siRNA against calcium and magnesium channels and transporters. Negative controls used were protein samples of untreated MCF-7 (Negative 1) and NP-Ca (Negative 2). siRNAs in combination were used to determine their synergistic downstream effect in the two pathways.

As shown in Figure 10, compared to the control (untreated or NP-treated cells), a lighter band of ~63 KDa was observed for phospho-AKT when siRNA targeting TRPC6 or a cocktail of siRNAs targeting TRPC6, TRPM8, and SLC41A1 were delivered into MCF-7 cells with CA NPs. Treatment with the same cocktail of three siRNAs even led to a decrease in total AKT (~62 KDa) level, suggesting possible cross-talk(s) of the AKT pathway with TRPC6. No interference was observed in the expression level of GAPDH. On the other hand, no significant change in either phospho- or total MAPK intensity was observed compared to the control for any of the treatments with TRPC6 siRNA or a group of siRNAs.

## 4. Conclusions

To our knowledge, this is the first report on the intracellular delivery of siRNA(s) with the help of inorganic nanocarriers targeting calcium ion channels and transporter genes that efficiently kills breast cancer cells. In summary, we found TRPC6, TRPM7, TRPM8, SLC41A1, SLC41A2, ORAI1, ORAI3, and ATP2C1 to be promising therapeutic targets for siRNA-mediated knockdown in breast cancer cells, with the simultaneous knockdown of TRPC6, TRPM8, SLC41A2, and MAGT1 through the CA NP-assisted combined delivery of respective siRNAs, resulting in a synergistic effect on cytotoxicity. Further studies in an animal model of breast cancer would pave the way to further explore the potentials of CA NPs for the intravenous delivery of siRNA(s) targeting calcium ion channel genes.

## Figures and Tables

**Table 1:** siRNAs used in this study.

Targeted Gene (Symbol)	Official Name
*ORAI 1*	ORAI Calcium Release-Activated Calcium Modulator 1
*ORAI3*	ORAI Calcium Release-Activated Calcium Modulator 3
*ATP2C1*	ATPase, Ca2+ Transporting, Type 2C, Member 1
*SLC41A1*	Solute Carrier Family 41 (Magnesium Transporter), Member 1
*SLC41A2*	Solute Carrier Family 41 (Magnesium Transporter), Member 2
*MAGT1*	Magnesium Transporter 1
*TRPM8*	Transient Receptor Potential Cation Channel, Subfamily M, Member 8
*TRPM7*	Transient Receptor Potential Cation Channel, Subfamily M, Member 7
*TRPV6*	Transient Receptor Potential Cation Channel, Subfamily V, Member 6
*TRPC6*	Transient Receptor Potential Cation Channel, Subfamily C, Member 6

**Table 2:** siRNA and their targeted gene sequences as sourced by Qiagen.

siRNA Targeted Gene Symbol	Target Gene Sequences
Allstars Negative control siRNA	Proprietary
*ORAI 1*	TCCGCTGTCCCGCTCCGGCTCCTGGGGCTC
*ORAI3*	GCTGGCGTGAGCTGGGGACGTTGCGGGCAC
*ATP2C1*	AGGAGTGCGGGGCGCGACTGGCGGCCGGC
*SLC41A1*	AGTGCTTGATGGGGCTGCCTGTTGGTGGAT
*SLC41A2*	ATCCAGTCCTTCTGTGGAACTTCTGAACAT
*MAGT1*	GTGTAGCGCCAGCGCGCTGTGACGTAATGT
*TRPM8*	CTCCCATGATGTCCTCACTGAACTCTTCTCC
*TRPM7*	GCGCCGCTCACGTGGTCCGTCCCAGCCCC
*TRPV6*	AGAGTCCTGGCTGGCTCTGCCAAGTGTAAC
*TRPC 6*	GGGATCTTGACGGAGAGTGCGGGGATGAA

**Table 3:** Particle size measurement of the CA NPs prepared in 200 µL Dulbecco’s modified Eagle medium (DMEM) media along with 15 mM of CaCl_2_ by Zetasizer and field emission scanning electron microscopy (FE-SEM).

Instruments Used	NP Size Measured (nM)
Zetasizer	191–284
FE-SEM	171–186

## Supplementary Materials

The following are available online at http://www.mdpi.com/2305-6304/6/3/34/s1, Figure S1: Calibration curve for fluorescence-labeled siRNA; Figure S2: Statistical analysis of cell viability following NPs-mediated intracellular delivery of selected siRNAs targeting cation transporters and channels.
