# Supplementary material for: Carbonate Apatite Nanoparticles-Facilitated Intracellular Delivery of siRNA(s) Targeting Calcium Ion Channels Efficiently Kills Breast Cancer Cells
Source: Toxics. 2018 Jun 26;6(3):34. doi: 10.3390/toxics6030034 (PMC6161028; doi:10.3390/toxics6030034)
Supplement: Supplementary file 1 [file toxics-06-00034-s001.pdf]

# Supplementary Materials: Carbonate Apatite Nanoparticles-Facilitated Intracellular Delivery of siRNA(s) Targeting Calcium Ion Channels Efficiently Kills Breast Cancer Cells

**Mohammad Borhan Uddin, Balakavitha Balaravi Pillai, Kyi Kyi Tha, Maeirah Ashaie, Md. Emranul Karim and Ezharul Hoque Chowdhury**

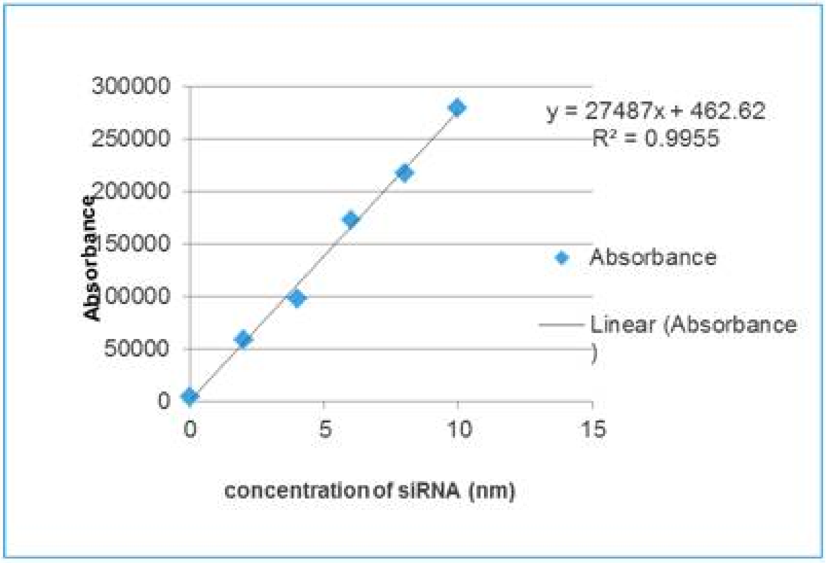


**Figure S1.** Calibration curve for fluorescence-labeled siRNA.

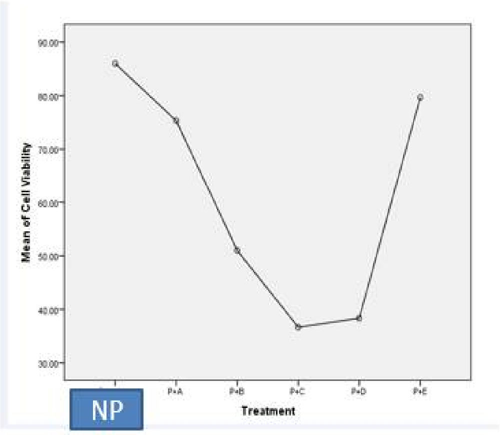


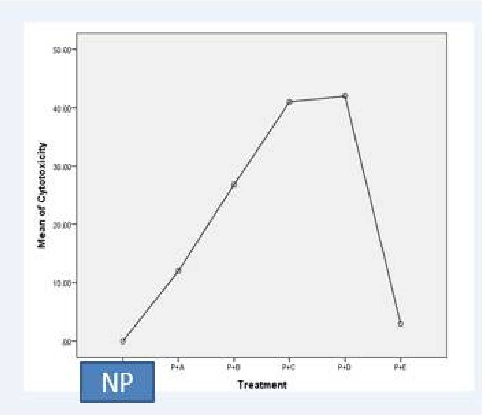


**ANOVA**

| | | Sum of Squares | df | Mean Square | F | Sig. |
|---|---|---|---|---|---|---|
| Cell Viability | Between Groups | 7153.833 | 5 | 1430.767 | 32.233 | .000 |
| | Within Groups | 532.667 | 12 | 44.389 | | |
| | Total | 7686.500 | 17 | | | |
| Cytotoxicity | Between Groups | 5160.606 | 5 | 1032.121 | 176107.733 | .000 |
| | Within Groups | .070 | 12 | .006 | | |
| | Total | 5160.676 | 17 | | | |

Values are significant (*) at p value 0.01 to 0.05,very significant (**) at p value 0.001 to 0.01, extremely significant (***) at p value 0.0001 to 0.001, And extremely significant (****) at p value < 0.0001.

| Treatment | Symbol (graph) |
|---|---|
| CA $Ca^{+2}$ 15 mM | NP |
| CA $Ca^{+2}$ 15 mM + ORAI1 | P+A |
| CA $Ca^{+2}$ 15 Mm + ATP2C1 | P+B |
| CA $Ca^{+2}$ 15 mM + TRPM7 | P+C |
| CA $Ca^{+2}$ 15 mM + ORAI3 | P+D |
| CA $Ca^{+2}$ 15 mM + AF 488 negative siRNA | P+E |

**Figure S2.** Statistical analysis of cell viability following NPs-mediated intracellular delivery of selected siRNAs targeting cation transporters and channels.
